# Reply to Wimalawansa, S.J. Comment on “Bertoldo et al. Definition, Assessment, and Management of Vitamin D Inadequacy: Suggestions, Recommendations, and Warnings from the Italian Society for Osteoporosis, Mineral Metabolism and Bone Diseases (SIOMMMS). *Nutrients* 2022, *14*, 4148”

**DOI:** 10.3390/nu15030499

**Published:** 2023-01-18

**Authors:** Francesco Bertoldo, Luisella Cianferotti, Marco Di Monaco, Alberto Falchetti, Angelo Fassio, Davide Gatti, Luigi Gennari, Sandro Giannini, Giuseppe Girasole, Stefano Gonnelli, Nazzarena Malavolta, Salvatore Minisola, Mario Pedrazzoni, Domenico Rendina, Maurizio Rossini, Iacopo Chiodini

**Affiliations:** 1 Emergency Medicine, Department of Medicine, University of Verona, 37129 Verona, Italy; 2 Bone Metabolic Diseases Unit, Department of Experimental, Clinical and Biomedical Sciences, University of Florence, 50121 Florence, Italy; 3 Osteoporosis Research Center, Fondazione Opera San Camillo, Presidio di Torino, 10131 Torino, Italy; 4 Laboratory of Experimental Clinical Research on Bone Metabolism, Istituto Auxologico Italiano, Istituto di Ricovero e Cura a Carattere Scientifico (IRCCS), 20145 Milan, Italy; 5 Rheumatology Unit, University of Verona, Policlinico GB Rossi, 37134 Verona, Italy; 6 Department of Medicine Surgery and Neuroscience, University of Siena, 53100 Siena, Italy; 7 Clinica Medica 1, Department of Medicine, University of Padova, 35122 Padova, Italy; 8 Rheumatology Department, “La Colletta” Hospital, ASL 3 Genovese, 16011 Arenzano, Italy; 9 Casa di Cura Madre Fortunata Toniolo, and Centri Medici Dyadea, 40141 Bologna, Italy; 10 U.O.C. Medicina Interna A, Malattie Metaboliche dell’Osso Ambulatorio Osteoporosi e Osteopatie Fragilizzanti, Sapienza University of Rome, 00185 Rome, Italy; 11 Department of Medicine and Surgery, University of Parma, 43121 Parma, Italy; 12 Department of Clinical Medicine, Surgery Federico II University, 80138 Naples, Italy; 13 Unit for Bone Metabolism Diseases and Diabetes, Istituto Auxologico Italiano, Istituto di Ricovero e Cura a Carattere Scientifico (IRCCS), 20133 Milan, Italy; 14 Department of Medical Biotechnology and Translational Medicine, University of Milan, 20122 Milan, Italy

With regard to Dr. Wimalawansa’s comment [1], his statement that defines the recommendations contained in the paper by Bertoldo F. et al. [2] as outdated as they do not consider the potential extra-skeletal positive effects of vitamin D. This statement represents his respectable point of view only, as our is and is more representative of the position of a scientific society called the “Italian Society for Osteoporosis, Mineral Metabolism and Bone Diseases”. In the abstract, it is clearly specified and easily readable: “Although vitamin D supplementation is also recommended by the Italian Medicine Agency for patients at risk for fragility fracture or for initiating osteoporotic medication, the therapeutic gap for osteoporosis in Italy is very high” which clearly delimits and defines our specific field of interest.

Dr. Wimalawansa’s main criticism of the recommendations of the Italian Society of Mineral Metabolism and Skeletal Diseases is centered on the fact that the use of vitamin D for extra-skeletal end points is not included in the recommendations. Consequently, this implies that its use in the otherwise healthy population without risk of low vitamin D levels is not recommended and that the threshold levels of administration and recommended dosage reflects only skeletal end points. This is explicitly stated point by point. The fact that potential benefits on extra-skeletal endpoints are not considered does not make these recommendations obsolete. At minimum, in Italy, the label use of cholecalciferol is based on “skeletal” effects and the recommendations focus on appropriateness to optimize these end points. In Italy, for example, there is still an important therapeutic gap in osteoporosis and vitamin D supplementation in combination with anti-fracture drugs. The SIOMMMS recommendations are also important in Italy where the regulatory agency for drug reimbursability [3], as a result of the significant growth in spending on vitamin D, has regulated reimbursability leading to a reduction in prescriptions of 30–34%. This also included a large portion of individuals requiring supplementation [4]. We hope that in the Italian population, where the prevalence of low vitamin D levels is still very high, our recommendations can make its prescription more appropriate.

In summary, we want to underline that:

- As stated and tasked by SIOMMMS, the recommendations concern only skeletal targets;
- The answers to his objections can be found in the text, as the result of the evaluation of the available evidence and of the sharing between experts;
- His comment confirms the opportunity of our article to avoid “personal opinion” and provide more supported and shared recommendations.
